# Sarcopenia em idosos com fratura do quadril: Estudo da prevalência, correlação com obesidade e ultrassonografia do reto femoral

**DOI:** 10.1055/s-0045-1813002

**Published:** 2025-12-15

**Authors:** Maurício Rodrigues Miyasaki, Juliano Casonatto, Carolina Morgato de Mello Miyasaki, Bruno Leguizamón Baruki, Yano Altomar de Sá, Rodrigo Antonio Carvalho Andraus

**Affiliations:** 1 Programa de Pós-Graduação em Ciências da Reabilitação, Universidade Pitágoras UNOPAR Anhanguera, Londrina, PR, Brasil; 2 Grupo de Pesquisa em Fisiologia e Atividade Física, Universidade Pitágoras UNOPAR Anhanguera, Londrina, PR, Brazil; 3 Faculdade de Medicina, Universidade Federal do Paraná, Curitiba, PR, Brasil; 4 Irmandade da Santa Casa de Londrina, Londrina, PR, Brasil; 5 Programa de Pós-Graduação em Movimento Humano e Reabilitação, Universidade Evangélica de Goiás (UniEVANGELICA), Anápolis, GO, Brasil

**Keywords:** fraturas do quadril, obesidade, sarcopenia, ultrassonografia, hip fractures, obesity, sarcopenia, ultrasonography

## Abstract

**Objetivo:**

Determinar a prevalência de provável sarcopenia em pacientes idosos com fraturas de quadril e avaliar a correlação entre as medidas ultrassonográficas do reto femoral, obesidade e diagnóstico de sarcopenia.

**Métodos:**

Sessenta e cinco participantes com idade ≥ 60 anos hospitalizados com fraturas de quadril foram incluídos. O questionário SARC-F foi aplicado e a circunferência da panturrilha e a força de preensão manual foram medidas. A espessura bilateral do reto femoral e a área de secção transversal foram avaliadas por ultrassonografia.

**Resultados:**

Provável sarcopenia foi identificada em 13 participantes (20,6%). A espessura média do reto femoral foi de 1,03 cm (desvio padrão [DP] = 0,22) para a coxa direita e 1,03 cm (DP = 0,23) para a coxa esquerda. A área de secção transversal média foi de 2,61 cm ^2^ (DP = 0,71) na coxa direita e 2,97 cm ^2^ (DP = 0,69) na coxa esquerda. A circunferência média da panturrilha foi de 31 cm (DP = 4,29) para a perna direita e 31 cm (DP = 4,31) para a esquerda. Não foram encontradas correlações entre as medidas ultrassonográficas do reto femoral e a sarcopenia provável. Indivíduos com sobrepeso e fratura de quadril apresentaram quatro vezes mais chances de apresentar o diagnóstico de sarcopenia provável.

**Conclusão:**

A prevalência de sarcopenia provável foi de 20,6%. Não foi observada correlação entre as medidas ultrassonográficas do reto femoral e sarcopenia. O sobrepeso esteve significativamente associado a um aumento de quatro vezes na probabilidade de sarcopenia provável nessa população.

## Abstract (en)

**Objective:**

To determine the prevalence of probable sarcopenia in senior patients with hip fractures and to evaluate the correlation between ultrasound measurements of the rectus femoris (RF) muscle, obesity, and the diagnosis of sarcopenia.

**Methods:**

The present study included 65 participants aged ≥ 60 years old admitted due to hip fractures. We administered the Strength, Assistance with Walking, Rising from a Chair, Climbing Stairs, and Falls (SARC-F) questionnaire, and measured the calf circumference and handgrip strength. We also assessed the thickness and cross-sectional area of the bilateral RF muscle using ultrasound.

**Results:**

We identified probable sarcopenia in 13 participants (20.6%). The mean RF thickness was 1.03 cm (standard deviation [SD] = 0.22) for the right thigh and 1.03 cm (SD = 0.23) for the left thigh. The mean cross-sectional area was 2.61 cm ^2^ (SD = 0.71) in the right thigh and 2.97 cm ^2^ (SD = 0.69) in the left thigh. The average calf circumference was 31 cm (SD = 4.29) for the right leg and 31 cm (SD = 4.31) for the left leg. We did not find correlations between ultrasound measurements of the RF muscle and potential sarcopenia. The diagnosis of probable sarcopenia was four times more likely in subjects who were overweight and had hip fractures.

**Conclusion:**

The prevalence of probable sarcopenia was 20.6%. There was no correlation between ultrasound measurements of the RF and the presence of sarcopenia. Overweight significantly increased fourfold the likelihood of probable sarcopenia in this population.

## Introdução

A sarcopenia no idoso é um grave problema de saúde pública pois está relacionada a aumento do risco de queda e fratura, 1 limita a capacidade de realizar atividades da vida diária, 2 está associada a doença cardíaca, respiratória e a comprometimento cognitivo, 3 leva a limitação de mobilidade relacionada a diminuição da velocidade da marcha 4 e contribui para diminuição da qualidade de vida, perda de independência ou necessidade de institucionalização. 3

A sarcopenia representa um fator significativo em pacientes com fraturas de quadril devido à sua associação tanto com os fatores etiológicos que contribuem para a fratura, como osteoporose e quedas, 5 6 quanto às suas complicações subsequentes. Adultos mais velhos que sofrem fraturas de quadril normalmente enfrentam longas internações hospitalares, 7 altas taxas de mortalidade em um ano 7 e maiores desafios na reabilitação, frequentemente resultando em limitações funcionais mais acentuadas.

Nas fraturas do quadril em idosos, há diversas publicações comparando técnicas cirúrgicas e tipos de implantes 8 e temos protocolos bastante confiáveis para planejar o tratamento cirúrgico. Os cuidados clínicos e a adoção de cuidados ortogeriátricos 9 com equipes multidisciplinares também garantem um melhor resultado cirúrgico. Porém, a sarcopenia, que é um fator que tem peso relevante na reabilitação e no prognóstico destes idosos, ainda não recebe a mesma atenção na abordagem destes indivíduos.

Os objetivos deste estudo são determinar a prevalência de sarcopenia em idosos internados com fratura do quadril, avaliar a correlação entre medidas ultrassonográficas do reto femoral e o diagnóstico de sarcopenia e verificar a correlação entre obesidade e o diagnóstico de sarcopenia.

As hipóteses deste estudo são que a população de idosos com fratura de fêmur apresenta uma alta prevalência de sarcopenia, e que tanto a espessura ultrassonográfica do reto femoral quanto a obesidade estão correlacionadas com o diagnóstico de sarcopenia.

## Métodos

O presente estudo possui caráter transversal e foi aprovado pelo comitê de ética da instituição (parecer 6.594.376; CAAE: 7365123.0.0000.0099).

Foram avaliados 65 participantes com ≥ 60 anos internados no período de 29 março de 2023 a 28 de março de 2024 com diagnóstico de fratura proximal do fêmur. Foram incluídos indivíduos com capacidade cognitiva para responder os questionários e que, junto com o familiar responsável, aceitaram participar do estudo. Excluímos pacientes com doença neoplásica, aqueles internados na unidade de terapia intensiva (UTI) antes da cirurgia e aqueles que apresentaram comprometimento cognitivo que os impediu de preencher o questionário autoaplicável SARC-F.

Os participantes foram convidados a integrar o estudo e foram avaliados antes do procedimento cirúrgico. Durante essa avaliação, foram coletados dados sobre comorbidades e o tipo de fratura, além de peso e altura, informados pelos participantes ou seus familiares, para o cálculo do índice de massa corpórea (IMC). Adicionalmente, todos responderam ao questionário SARC-F.

**Fig. 1:**
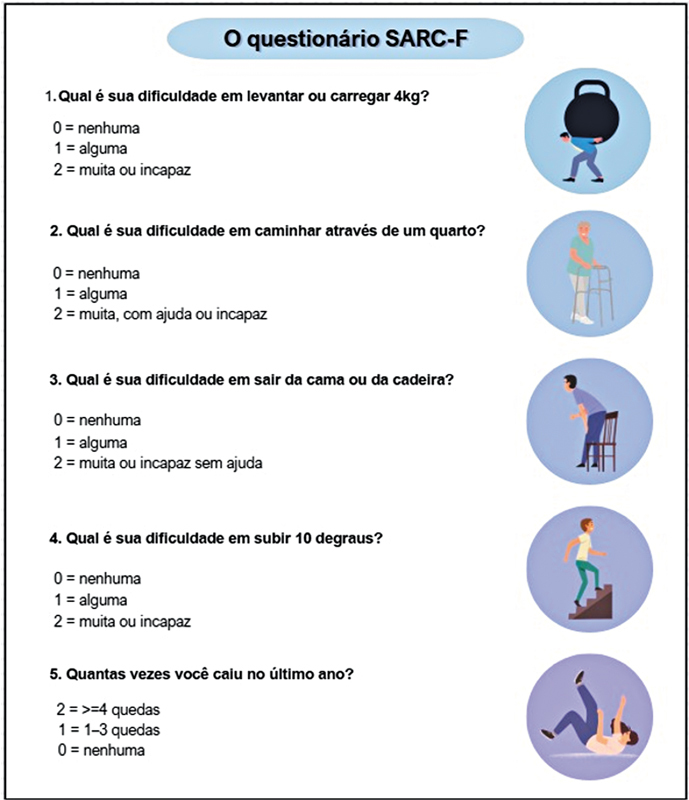
Questionário Strength, Assistance with Walking, Rising from a Chair, Climbing Stairs, and Falls (SARC-F).

O SARC-F ( Fig. 1 ) é um questionário autorrelatado, composto por cinco itens, elaborado para rastrear o risco de sarcopenia. Ele avalia a percepção do indivíduo sobre suas limitações em áreas funcionais importantes, incluindo força muscular, capacidade de caminhar, capacidade de levantar-se de uma cadeira, de subir escadas e histórico de quedas. A versão traduzida para o português foi validada no Brasil por Barbosa-Silva et al. 10

Foi aplicado teste de força de preensão palmar com um aparelho portátil digital Instrutherm DM-90 (Instrutherm). O teste foi realizado com o participante deitado e o antebraço no lado dominante apoiado ao longo do corpo. Foram realizadas três tentativas com intervalo de um minuto e foi anotado o maior valor obtido. Com uma fita métrica, foi medida a circunferência das panturrilhas 2 cm abaixo da tuberosidade anterior da tíbia com o participante na posição deitada. Foi realizada a medida da espessura e da área de secção transversal do músculo reto femoral bilateral através da realização de ultrassonografia com aparelho Philips Lumify (Royal Philips Inc.) e transdutor linear em um ponto de coxa equidistante entre a espinha ilíaca anterossuperior e o polo superior da patela. O examinador foi um único ortopedista com > 5 anos de experiência de utilização da ultrassonografia na prática clínica e que estava cego para o diagnóstico de sarcopenia.

### Variável sarcopenia

**Fig. 2:**
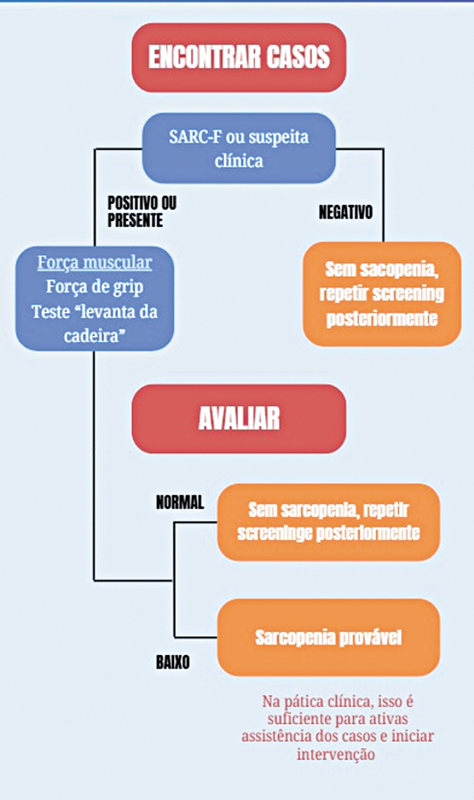
Algoritmo com os dois primeiros passos para o de diagnóstico de sarcopenia (European Work Group on Sarcopenia in Older People revisados em 2019 - EWGSOP2).

Para se determinar o diagnóstico de sarcopenia provável, foram utilizados os critérios do European Work Group on Sarcopenia in Older People revisados em 2019 (EWGSOP2) 3 ( Fig. 2 ). Para o diagnóstico de sarcopenia provável, foi considerada a pontuação no SARC-F > 4 e força de flexão palmar < 27 kg para homens e < 16 kg para mulheres.

### Massa muscular

A massa muscular total foi estimada pela equação desenvolvida por Lee et al. 11 (massa muscular total = 0,244 x peso + 7,80 x altura + 6,6 x sexo - 0,098 x idade + raça - 3,3). O peso corporal foi utilizado em quilogramas, a estatura em metros e a idade em anos. Foi atribuído 0 para mulheres e 1 para homens, para asiáticos -1,2, para negros 1,4 e 0 para brancos. Esta equação foi validada para a população brasileira usando densitometria de composição corporal (DEXA, do inglês *dual-energy x-ray absorptiometry* ) como “padrão ouro” e sua correlação com a equação foi de R = 0,86 para homens e R = 0,90 para mulheres. O valor preditivo entre a DEXA e a equação foi considerado forte (K = 0,74; *p* < 0,001) com alta sensibilidade (89%) e especificidade (86%). 11 A equação foi ajustada dividindo pela estatura ao quadrado, criando um índice de massa muscular total. O ponto de corte utilizado para determinar uma reduzida massa muscular foi ≤ 5,75 kg/m ^2^ para mulheres e ≤ 8,50 kg/m ^2^ para homens. 12

A estimativa de massa muscular foi calculada para investigar a correlação com o diagnóstico de sarcopenia e com as medidas ultrassonográficas do músculo reto femoral.

### Análise dos dados

As análises estatísticas foram realizadas no software IBM SPSS Statistics for Windows, (IBM Corp.), versão 22.0, adotando-se um nível de significância de 5% ( *p* ≤ 0,05) e um intervalo de confiança (IC) de 95%.

Inicialmente, a normalidade das variáveis contínuas foi avaliada utilizando o teste de Shapiro-Wilk, por ser mais adequado para amostras pequenas a moderadas. Com exceção da variável área do reto femoral esquerdo, todas apresentaram distribuição normal, compatível com os pressupostos dos testes paramétricos. Além disso, foram observadas a independência entre as amostras e a homogeneidade das variâncias por meio da inspeção do teste de Levene, o que reforça a adequação da utilização do teste *t* para amostras independentes nas comparações entre os grupos (eutróficos *versus* excesso de peso).

Para a variável área do reto femoral esquerdo, que apresentou distribuição não paramétrica, foi aplicado o teste de Mann-Whitney.

O teste do Qui-quadrado foi empregado para investigar associações entre variáveis categóricas. Para a análise da magnitude dessas associações, utilizou-se regressão logística binária, com cálculo das razões de chances (RCs) e seus respectivos IC95%.

## Resultados

O presente estudo foi composto por 65 participantes, sendo 22 do sexo masculino e 43 do sexo feminino. A média de idade foi de 77 anos, variando de 61 a 93 anos. A média do IMC foi de 24,5 (DP = 5,6), sendo que 13 participantes foram considerados com sobrepeso (IMC ≥ 25). O diagnóstico da fratura foi de fratura do colo do fêmur em 38 e de fratura pertrocantérica em 27 participantes.

A pontuação obtida no questionário SARC-F foi maior ou igual a 4 para 16 indivíduos e a média da força de preensão palmar foi de 18,8 kg (DP = 7,4). Aplicando os critérios do EWGSOP2, 3 identificamos 13 pacientes com diagnóstico provável de sarcopenia (20,6% da amostra), dos quais 9 (69,2%) eram do sexo feminino e 4 (30,8%) do sexo masculino.

A média encontrada da espessura do reto femoral medida pela ultrassonografia foi de 1,03 cm (DP = 0,22) para a coxa direita e 1,3 (DP = 0,23) para a esquerda. A média da área de secção transversal do músculo reto femoral foi de 2,61 cm ^2^ à direita e 2,97 cm ^2^ à esquerda. A medida da circunferência da panturrilha obteve a média de 31 cm nos membros inferiores direito e esquerdo. A medida mínima foi de 24 cm bilateralmente e a máxima foi de 42 cm à direita e 44 cm à esquerda. O cálculo do índice de massa muscular através da aplicação da fórmula de Lee obteve um resultado médio de 6,7 kg/m ^2^ para mulheres e de 8,8 kg/m ^2^ para homens. A análise estatística não demonstrou correlação entre os resultados de índice de massa muscular, idade, circunferência da panturrilha e medidas do músculo reto femoral por ultrassonografia com o diagnóstico provável de sarcopenia ( Tabela 1 ). Entretanto, observou-se que os indivíduos com provável sarcopenia apresentaram uma média de IMC significativamente maior ( *p* = 0,046) e menor força de preensão palmar ( *p* = 0,007).

**Tabela 1:** Comparação de variáveis antropométricas, funcionais e musculares entre idosos com e sem sarcopenia provável após fratura do quadril

Variável	Sarcopenia provável	*n*	média	DP	valor-t	Valor de *p*
Índice de massa muscular (kg/m ^2^ )	Não	50	19	5,6	−1,468	0,147
	Sim	13	21	6,8		
Idade (anos)	Não	50	76	1,1	−0,856	0,395
	Sim	13	78	2,2		
IMC (m/cm ^2^ )	Não	50	23	0,613	−2,040	0,046
	Sim	13	27	2,482		
Força de preensão palmar (kg)	Não	50	20	7,3	2,770	0,007
	Sim	13	14	5,4		
Circunferência da panturrilha (cm)	Não	50	31	5,2	−0,190	0,850
	Sim	13	32	41,1		
Espessura do reto femoral (cm)	Não	50	1,020	0,218	−0,542	0,590
	Sim	13	1,095	0,225		
Área de secção transversal do reto femoral (cm ^2^ )	Não	50	2,569	0,672	−1,194	0,237
	Sim	13	2,832	0,841		

**Abreviações** : cm, centímetros; cm ^2^ , centímetros quadrados; DP, desvio padrão; IMC, índice de massa corporal; kg/m ^2^ , quilogramas por metro quadrado (unidade do IMC e do índice de massa muscular); n, número de participantes; Valor de *p* : nível de significância estatística; Valor de *t* : valor da estatística t do teste t de Student para amostras independentes.

Também encontramos associação entre a probabilidade de sarcopenia e o excesso de peso (sobrepeso e obesidade) ( Tabela 2 ). A regressão logística mostrou grande magnitude da associação, sendo que a RC encontrada foi de 4,235 (IC95% = 1,135–15,799; *p* = 0,032).

**Tabela 2:** Associação entre estado nutricional e sarcopenia provável em idosos com fratura do quadril

		Estado nutricional						
		Normal		Excesso				
		*n*	%	*n*	%	X ^2^	Valor de *p*	Phi
Sarcopenia provável	Não	32	88,9%	17	65,4%	5,033	0,025	0,285
	Sim	4	11,1%	9	34,6%			
Total		36	100,0%	26	100,0%			

**Abreviações** : n, número de participantes; %, porcentagem; X ^2^ , valor da estatística do teste do Qui-quadrado de Pearson; Valor de *p* , nível de significância estatística; Phi, coeficiente de associação de Phi, usado para medir a força da associação entre variáveis categóricas.

Não encontramos correlação entre excesso de peso e a força de preensão palmar, ou medida ultrassonográfica da área e espessura do reto femoral. Contudo, indivíduos com excesso de peso apresentaram maior circunferência da panturrilha ( Tabela 3 ).

**Tabela 3:** Indicadores de sarcopenia em idosos com fratura do quadril: comparação entre eutróficos e com excesso de peso

	Estado nutricional	*n*	Média	DP	Valo *r* de *t*	Valor de *p*
Preensão manual (kg)	Eutrófico	36	19,85	7,18	0,460	0,647
	Excesso de peso	26	18,96	7,98		
Circunferência da panturrilha(cm)	Eutrófico	27	29,3	3,8	−4,433	< 0,001
	Excesso de peso	22	34,3	3,8		
Espessura RF_direita	Eutrófico	36	1,00	0,20	−1,461	0,149
	Excesso de peso	26	1,08	0,24		
Espessura RF_esquerda	Eutrófico	36	1,01	0,21	−0,865	0,390
	Excesso de peso	26	1,06	0,26		
Area RF_direita	Eutrófico	36	2,48	0,64	−1,959	0,055
	Excesso de peso	26	2,83	0,75		
	Estado nutricional	*n*	Mediana	II	Z	Valor de *p*
Area RF_esquerda	Eutrófico	36	2,45	1,06	−1,405	0,160
	Excesso de peso	26	2,80	1,04		

**Abreviações** : n, número de participantes; DP, desvio padrão; II, intervalo interquartílico; Valor de *p* , nível de significância estatística; RF, reto femoral; Valor de *t* , valor da estatística t do teste t de Student para amostras independentes; Z, valor da estatística Z do teste de Mann-Whitney.

## Discussão

O algoritmo de diagnóstico de sarcopenia proposto pelo EWGSOP2 3 tem quatro etapas ( Fig. 1 ). Nas duas primeiras, ele utiliza os critérios de baixa pontuação no questionário SARC-F e baixa força muscular para estabelecer o diagnostico provável de sarcopenia. O terceiro passo do algoritmo é a confirmação do diagnóstico de sarcopenia, quando são utilizados exames para quantificar a massa muscular incluindo a DEXA, a tomografia computadorizada (TC) ou o exame de ressonância magnética (RM) da coxa. Para a avaliação de indivíduos idosos, a DEXA apresenta vantagens importantes, como pouca exposição à radiação e mínima necessidade de cooperação do paciente. 13

Como na prática clínica, com o paciente internado para tratar a fratura, raramente a utilização destes recursos está à disposição do cirurgião, optamos por parar no segundo passo do algoritmo e separar a amostra em indivíduos com ou sem o diagnóstico de sarcopenia provável. De acordo com o EWGSOP2, a identificação de sarcopenia provável é considerada suficiente para o início da investigação de suas causas e para a implementação de intervenções clínicas. Portanto, ao focarmos na sarcopenia provável, garantimos a validade do diagnóstico para o objetivo do nosso estudo, que é a identificação precoce dessa condição em um cenário de prática clínica. Consideramos que a não progressão para as etapas subsequentes do algoritmo limita a capacidade de classificar a gravidade da sarcopenia, mas não a acurácia da sua identificação inicial, que foi o foco da nossa pesquisa.

Embora o consenso sobre a definição e os critérios de diagnóstico tenha evoluído, 14 ainda há dificuldade na comparação dos resultados de prevalência de sarcopenia em idosos, que variam de acordo com os critérios adotados para a definição da condição e dos pontos de corte utilizados e métodos de medida de força e massa muscular. 15

Na metanálise de Nascimento et al. 16 o resultado de prevalência na população geral foi de 10%, Na metanálise de Petermann-Rocha et al., 17 o mesmo resultado foi de 16%. Apesar de que nos dois estudos a população analisada era de idosos saudáveis, os resultados mostraram variação considerável, principalmente entre os artigos que utilizavam diferentes critérios de diagnóstico. O estudo Saúde, Bem-Estar e Envelhecimento (SABE), 18 conduzido com idosos residentes na cidade de São Paulo, obteve um resultado de prevalência de sarcopenia de 16,1% para mulheres e 14% para homens. A prevalência de sarcopenia é diferente em grupos de pacientes com condições específicas quando comparados à população geral, sendo relatada de 18% em pacientes diabéticos a 66% em pacientes com câncer de esôfago. 15 A prevalência de sarcopenia em idosos com fratura do quadril encontrada na literatura variou de 17 a 37%. 19 20 Obtivemos o diagnóstico de sarcopenia provável em 20,2% de nossos pacientes, o que parece ser um resultado mais alto se comparado com idosos brasileiros saudáveis e semelhante ao obtido em outros estudos com idosos com fratura do quadril.

O teste de força de flexão palmar com dinamômetro é o teste preconizado pelo EWGSOP2 aplicável à nossa amostra de indivíduos com fratura dos membros inferiores. Este teste tem boa correlação com a força dos membros inferiores, é de fácil aplicação clínica, é utilizado em boa parte das publicações sobre o assunto e tem pontos de corte mais bem definidos para o sexo feminino e masculino. 3 21 Embora a avaliação da força de preensão palmar tenha sido realizada utilizando pontos de corte específicos para cada sexo, o tamanho da amostra do presente estudo não permitiu a realização de análises estratificadas por sexo para a prevalência de sarcopenia ou para a avaliação das associações com obesidade e ultrassonografia.

Os métodos para a determinação da massa muscular considerados para o diagnóstico de sarcopenia, como a RM, a densitometria ou a bioimpedância são caros ou pouco práticos para a aplicação em pacientes internados com uma condição aguda. Neste contexto, a ultrassonografia tem sido apontada como uma alternativa prática e acessível para avaliação da massa e da qualidade muscular. 22 Uma recente metanálise encontrou moderada acurácia diagnóstica para sarcopenia com a utilização da ultrassonografia para medir a espessura do músculo reto femoral em idosos saudáveis. Os dados compilados de 5 estudos apresentaram uma sensibilidade de 72% e especificidade de 72%. 23 A ultrassonografia como ferramenta de avaliação para sarcopenia tem sido defendida para a utilização em indivíduos internados com condições clínicas críticas. 24 Para idosos com fratura do quadril, um estudo encontrou correlação entre a espessura do quadríceps (reto femoral e vasto intermédio) e o diagnóstico de sarcopenia. 25 Em contraste com estes dados, não encontramos em nossa amostra correlação entre a área de secção transversa e a espessura do reto femoral medida pela ultrassonografia com o diagnóstico de sarcopenia provável, IMC, força de preensão palmar, circunferência da panturrilha ou com o índice de massa muscular. O estudo de Yamada et al. 26 sugere que a medida de espessura muscular está relacionada ao volume muscular e a avaliação da ecogenicidade está relacionada a função deste músculo. 26 No entanto, medidas ultrassonográficas de qualidade muscular como ecogenicidade, avaliação dos ângulos das fibras, elasticidade e vascularização são mais complexas e possivelmente não acessíveis ao cirurgião que efetivamente irá tratar o paciente.

A falta de correlação observada pode ser decorrente de limitações na metodologia ultrassonográfica. Primeiramente, há considerável heterogeneidade na literatura em relação aos protocolos de exame, particularmente no que diz respeito ao posicionamento do paciente e aos pontos de referência anatômicos. 23 Outro fator potencialmente influenciador é que nossas medições não foram realizadas por um especialista em ultrassonografia, visto que o objetivo principal era avaliar a viabilidade do uso dessa ferramenta na prática clínica de rotina, ou seja, avaliar a ferramenta nas mãos do médico assistente que, embora seja um ortopedista com experiência em ultrassonografia, não é especialista em radiologia.

Os músculos dos membros inferiores são os mais comumente avaliados para o diagnóstico ultrassonográfico de sarcopenia, provavelmente por serem mais fáceis de mensurar e mais diretamente associados à mobilidade e às atividades da vida diária em comparação com os músculos do tronco ou da cabeça. 23 Além disso, a avaliação ultrassonográfica do músculo masseter tem sido associada ao risco de disfagia, enquanto a avaliação do bíceps braquial tem sido associada à capacidade de realizar a autoalimentação. 25

Observamos grande heterogeneidade nos estudos citados com relação às características dos pacientes, locais e músculos examinados e principalmente com relação ao critério diagnóstico utilizado para a sarcopenia, o que torna difícil a comparação. Embora o consenso do EWGSOP2 reconheça que a ultrassonografia demonstra boa validade para a determinação da massa muscular, ele ressalta que mais pesquisa é necessária para validar o método para condições clínicas específicas. Essa ressalva se deve, em grande parte, a desafios como a falta de pontos de corte padronizados para o diagnóstico de sarcopenia via ultrassom, a heterogeneidade das técnicas e locais de exame (membros superiores *versus* inferiores, diferentes músculos) entre os estudos, e a limitada correlação observada até o momento entre as medidas ultrassonográficas e desfechos clínicos relevantes. Tais fatores dificultam a aplicação universal e a interpretação padronizada da ultrassonografia para o diagnóstico de sarcopenia na prática clínica.

Encontramos forte correlação positiva entre excesso de peso (IMC > 24,9) com o diagnóstico de sarcopenia provável. No entanto, alguns fatores de confusão devem ser considerados. Nossa análise não levou em conta variações específicas de sexo no declínio da massa muscular ou diferenças na distribuição de gordura entre grupos étnicos. Além disso, a obesidade em si é um fator de risco independente para quedas e fraturas em idosos. Todavia, este achado faz sentido se considerarmos que não encontramos em nossa amostra diferenças significantes nem na força muscular e nem na espessura do reto femoral em indivíduos com peso normal e os com excesso de peso. O envelhecimento é associado a um aumento proporcional de gordura e mudança de sua distribuição no corpo e essas alterações parecem estar conectadas do ponto de vista patogenético. 27

Para uma compreensão mais aprofundada da associação encontrada entre o excesso de peso e a sarcopenia provável, caracterizamos o perfil demográfico e funcional do subgrupo de participantes com excesso de peso e diagnóstico de sarcopenia provável ( *n* = 9). Neste grupo, a distribuição por sexo foi de 78% feminino e 22% masculino. A média da força de preensão manual foi de 13,4 ± 6 kg, e a pontuação média no questionário SARC-F foi de 6 ± 2 pontos, indicando limitação nas atividades diárias. Estes dados complementam a Tabela 2 , oferecendo um panorama mais detalhado das características clínicas e funcionais dos indivíduos com excesso de peso que também apresentam sarcopenia provável em nossa amostra. A média de IMC dos indivíduos sarcopênicos foi de 27 ± 2 Kg e a dos não sarcopênicos de 23 ± 0,6 Kg. A grande diferença de magnitude de DP pode se dever ao tamanho da amostra.

No presente estudo, o grupo de indivíduos com provável sarcopenia apresentou uma média de IMC de 27,0 kg/m ^2^ , superior à média de 23,0 kg/m ^2^ observada no grupo não sarcopênico. Contudo, chama a atenção a maior dispersão dos dados de IMC no grupo sarcopênico, evidenciada por um DP de 2,482, comparado ao DP de 0,613 no grupo não sarcopênico. Essa elevada heterogeneidade pode ser atribuída à complexidade fenotípica da sarcopenia, uma condição que pode coexistir com o excesso de peso (caracterizando a sarcopenia obesogênica) ou manifestar-se em indivíduos com peso normal ou baixo. Adicionalmente, o pequeno tamanho da amostra, especialmente no grupo com provável sarcopenia ( *n* = 13), pode ter influenciado este elevado DP, uma vez que amostras reduzidas são mais suscetíveis à influência de valores extremos e à representatividade limitada da variabilidade populacional.

A obesidade é frequentemente associada à inflamação crônica de baixo grau, que pode acelerar a degradação muscular e promover a resistência à insulina. Este estado inflamatório pode impactar negativamente a síntese de proteína muscular, exacerbando ainda mais a perda muscular. Além disso, o excesso de adiposidade induz alterações hormonais que afetam o metabolismo muscular. Níveis elevados de adipocinas, como leptina e interleucina-6, podem prejudicar a função muscular e contribuir para a progressão da sarcopenia. 27

A obesidade e a sarcopenia são duas condições que aumentam por si só as limitações funcionais e a morbidade em idosos. A associação de um terceiro fator associado a alta morbimortalidade como a fratura do quadril certamente aumenta as complicações do tratamento e merece atenção também do ponto de vista da prevenção com atenção à alimentação e à atividade física.

Embora o presente estudo não tenha realizado cálculo prévio do tamanho amostral, buscou-se mitigar esta limitação por meio da apresentação dos ICs e da interpretação dos tamanhos de efeito, como forma de reforçar a transparência e a robustez das análises. A regressão logística revelou uma associação estatisticamente significativa entre o excesso de peso e o diagnóstico provável de sarcopenia, com uma RC de 4,235 e IC95% entre 1,135 e 15,799 ( *p* = 0,032), indicando não apenas significância estatística, mas também uma magnitude de efeito clínica relevante. Apesar de os ICs relativamente amplos refletirem o tamanho reduzido da amostra, os achados sugerem uma associação consistente. Além disso, a diferença na força de preensão palmar entre os grupos com e sem sarcopenia provável foi estatisticamente significativa ( *p* = 0,004), com médias de 20,7 kg e 14,6 kg, respectivamente, correspondendo a um tamanho de efeito moderado a alto ( *d* de Cohen ∼ 0,89), o que reforça sua relevância funcional. Reconhece-se, no entanto, que o número relativamente pequeno de participantes pode ter limitado o poder estatístico das comparações, especialmente daquelas envolvendo medidas ultrassonográficas, aumentando o risco de erro do tipo II. Assim, recomenda-se que estudos futuros com amostras maiores sejam conduzidos para confirmar estes achados e ampliar a generalização dos resultados. Estas estratégias analíticas, contudo, oferecem ao leitor uma compreensão mais completa da magnitude e relevância clínica das associações observadas, mesmo diante das limitações amostrais.

## Conclusão

A prevalência de sarcopenia provável em nossa casuística foi de 20,6%, e não encontramos correlação entre medidas ultrassonográficas do reto femoral e o diagnóstico de sarcopenia provável. Indivíduos com fratura de quadril e sobrepeso apresentaram quatro vezes mais chances de apresentar diagnóstico provável de sarcopenia
